# Correlation of neomycin, faecal neutral and acid sterols with colon carcinogenesis in rats

**DOI:** 10.1038/sj.bjc.6690476

**Published:** 1999-06

**Authors:** S K Panda, S C Chattoraj, S A Broitman

**Affiliations:** Department of Chemistry, Maharaja Manindra Chandra College, Calcutta , 700003, India; Departments of Biochemistry and Microbiology, Boston University School of Medicine, 80 East Concord Street, MA, 02118, USA

**Keywords:** safflower oil, neomycin, faecal coprostanol, colon cancer

## Abstract

High fat diets have been implicated in incidence of colon cancer both in epidemiological and animal studies. Present investigation deals with the incidence, location and numbers of large and small bowel tumours induced by 1,2-dimethyl hydrazine (DMH) in rats fed high fat diets and neomycin. Neomycin was used to modify the faecal sterol metabolism and the relationship of the high fat diet and faecal neutral and acid sterols to the large bowel tumorigenesis was evaluated. DMH administered rats were fed with (a) 20% safflower oil; (b) 20% safflower oil and neomycin; (c) 20% safflower oil, cholesterol and cholic acid; and (d) 20% safflower oil, cholesterol, cholic acid and neomycin. Neomycin was found to be associated with both increase and decrease of tumour numbers. The faecal sterols lithocholic and deoxycholic acids were found to have no participation, while cholesterol and cholic acid were found to decrease with increase in tumour numbers. However, faecal coprostanol has been found to have a significant positive correlation with tumorigenesis in all dietary groups. Therefore coprostanol might possibly be associated with colon carcinogenesis in DMH-fed rats and cholesterol metabolism in gut appears to be related to the development of tumours. © 1999 Cancer Research Campaign


*British Journal of Cancer* (1999) **80**(8), 1132–1136
© 1999 Cancer Research Campaign
Article no. bjoc.1999.0476

### Correlation of neomycin, faecal neutral and acid sterols with colon carcinogenesis in rats

**SK Panda[1], SC Chattoraj[2] and SA Broitman[2]**

[1]Department of Chemistry, Maharaja Manindra Chandra College, Calcutta 700003, India; [2]Departments of Biochemistry and Microbiology, Boston University School of Medicine, 80 East Concord Street, MA 02118, USA

**Summary** High fat diets have been implicated in incidence of colon cancer both in epidemiological and animal studies. Present investigation deals with the incidence, location and numbers of large and small bowel tumours induced by 1,2-dimethyl hydrazine (DMH) in rats fed high fat diets and neomycin. Neomycin was used to modify the faecal sterol metabolism and the relationship of the high fat diet and faecal neutral and acid sterols to the large bowel tumorigenesis was evaluated. DMH administered rats were fed with (a) 20% safflower oil; (b) 20% safflower oil and neomycin; (c) 20% safflower oil, cholesterol and cholic acid; and (d) 20% safflower oil, cholesterol, cholic acid and neomycin. Neomycin was found to be associated with both increase and decrease of tumour numbers. The faecal sterols lithocholic and deoxycholic acids were found to have no participation, while cholesterol and cholic acid were found to decrease with increase in tumour numbers. However, faecal coprostanol has been found to have a significant positive correlation with tumorigenesis in all dietary groups. Therefore coprostanol might possibly be associated with colon carcinogenesis in DMH-fed rats and cholesterol metabolism in gut appears to be related to the development of tumours.

**Keywords:** safflower oil; neomycin; faecal coprostanol; colon cancer

Cancer of the large bowel has been the subject of intensive investigations for many years and its incidence has been found to vary with geographic area and socioeconomic level (Dolls, 1969). From the distribution of large bowel cancer in various parts of the world and data dealing with migrant populations, it is apparent that environmental factors, rather than genetic and social factors, play a significant role in the aetiology of colon cancer in man (Haenzel et al, 1973; Wynder, 1975).

Epidemiological studies including metabolic parameters of role of diet on colon cancer have revealed that populations consuming high fat diets have an increased risk of developing colon cancer compared to populations consuming vegetarian diets (Reddy et al, 1977; Cohen et al, 1978; Reddy, 1992). Animal model studies have shown that rats fed unsaturated fats developed more tumours than those fed saturated fats (Broitman et al, 1977; Carroll, 1987). Epidemiological studies have also shown that high-risk populations have increased concentrations of intestinal anaerobic bacteria and their enhanced metabolism of neutral sterols and primary bile acids in gut (Reddy, 1992; Corpet et al, 1994). The bacterial transformation of cholesterol to coprostanol and coprostanone in the large bowel was extremely high for the American population, comprising over 80% of the faecal sterols. Further studies have indicated that faecal excretions of cholesterol and/or its metabolites and bile acids were higher in patients with colorectal cancer and adenomatous polyps (Reddy et al, 1977).

Recent studies have implicated polyunsaturated fats rich in omega-6-polyunsaturated fatty acids (PUFAs) in the increased incidence of colon cancer (Cannizzo and Broitman, 1989; Lindner, 1991; Narisawa et al, 1991; Reddy, 1992; Narisawa et al, 1994; Ochiai et al, 1996) and have focused on the possibility that dietary fats exert a promoter effect subsequent to the initiation of induction by a carcinogen. The focal point is upon the faecal excretion of endogenously synthesized neutral and acid sterols, and their subsequent modification by the gut flora to carcinogens, promoters or co-carcinogenic agents (Reddy, 1992). Studies from this laboratory supported the observations that dietary lipid-induced alterations in the flora increase the production of carcinogenic metabolites, and that cholesterol metabolism in gut has a key role to play in this regard (Kraus et al, 1987; Fabricant and Broitman, 1990; Cerda et al, 1995).

The present study has been designed in an attempt to correlate the faecal concentration of one or more neutral and acid sterols, to the incidence of colon tumorigenesis. Neomycin, which is known to have a significant effect upon sterol metabolism in gut and their faecal concentration (Sedaghat et al, 1975), has been administered orally to rats fed polyunsaturated fat (safflower oil) and cholesterol diets. The influence of neomycin on the location, incidence and number of large and small bowel tumorigenesis in rats induced by DMH, and subsequent changes in faecal excretion of neutral and acid sterols have been investigated.

*Received 1 May 1998*
*Revised 5 January 1999*
*Accepted 6 January 1999*

*Correspondence to:* SK Panda

### MATERIALS AND METHODS

### Animals and diets

Sprague Dawley CD male weanling rats 30–35 days old, weighing 25–40 g (obtained from Charles River Breeding Laboratories, MA, USA) were randomized and divided into four groups (viz. I, I-N, II, II-N). Each group consisted of 20 rats and was fed the basal diet listed in Table 1 supplemented with the following:

I – 20% safflower oil diet (basal diet);
I-N – 20% safflower oil diet and neomycin sulphate 21 mg% in drinking water;
II – 20% safflower oil diet, 1% cholesterol and 0.3% cholic acid;

II-N – 20% safflower oil diet, 1% cholesterol, 0.3% cholic acid and neomycin sulphate 21 mg% in drinking water.

Neomycin was administered to rats in two dietary groups, I-N and II-N, as neomycin sulphate in their drinking water. The drinking water of the animals in dietary groups I and II did not contain any neomycin sulphate.

In Western diets, the principal PUFAs that effectively lower serum cholesterol levels are linoleic (C 18:2) and linolenic (C 18:3) acids. Safflower oil has been used in this experiment because it is rich in these PUFAs (77.4%). Cholic acid was used in the high cholesterol diet (II and II-N) in order to maintain a prolonged hypercholesterolaemia in rats. The vitamin powder and salt mixture provided the remaining known vitamins and minerals for adequate maintenance of the animals, and has been used previously in this laboratory (Broitman et al, 1977). The sucrose level was adjusted on a weight basis to compensate for addition or omission of nutrients from the basal diet. The 20% fat diets provided 36% of calories as fat and 4.53 Kcal $g^{-1}$ diet. After 2 weeks on the diet, each group received 10 mg $kg^{-1}$ of DMH dissolved in saline and injected intramuscularly once each week for 15 weeks. These were designated ID, IDN, IID and IIDN, respectively, and were maintained on the diets for an additional 20 weeks following the last DMH injections.

Animals were housed in individual cages in carefully controlled quarters at a constant temperature of 70 ± 2°F. They were weighed twice weekly, and at 2-week intervals stool examinations were performed to detect gastrointestinal tract bleeding. Food containers were weighed before and after each filling (on alternate days) to determine food consumption. Animals that 'spilled' diets were not included in the average for food intake. Animals were checked daily for physical appearance and moribund animals noted. Animals that died prior to the termination date were autopsied. Kidneys, lungs, liver, spleen and testes were removed from all rats and examined in the gross. The stomach, with the oesophagus intact, small and large bowel were washed with ice-cold saline and then formaldehyde, and examined under a 10× magnification lens for the presence of lesions. Lesions in the gross were verified by routine histological procedures, i.e. staining with haematoxylin, eosin and periodic acid-Schiff stains. The remaining portions of the hollow viscera in which lesions were not apparent grossly, were rolled, sectioned and examined histologically.

**Table 1** Composition of polyunsaturated fat diet[a] (basal diet)

| Constituents | Percentage composition of 20% fat diet |
|---|---|
| Safflower oil | 20.0 |
| Casein (vitamin-free) | 18.0 |
| Sucrose | 51.6 |
| Choline chloride | 0.3 |
| Salt mixture (hegsted) | 4.0 |
| Vitamin mixture | 1.0 |
| Vitamin A, D, E mix | 0.1 |
| Alphacel | 5.0 |
| | 100.0 |
| Kcal/g (calculated) | 4.53 |

[a]Salt mixture (Hegsted) obtained from ICN Pharmaceuticals Inc (Cleveland, OH, USA). Vitamin composition of diets as reported in Broitman *et al* (1977).

**Table 2** Food consumption in rats fed various dietary regimens with and without neomycin

| | Food consumption (g per day) | | |
|---|---|---|---|
| Dietary group | Week 4 | Week 16 | Week 32 |
| ID | 15.0 ± 1.4[a] | 18.7 ± 0.4 | 18.1 ± 0.6 |
| IDN | 18.0 ± 0.7 | 16.6 ± 1.0 | 19.1 ± 1.0 |
| IID | 21.6 ± 1.7 | 18.0 ± 1.2 | 15.5 ± 1.6 |
| IIDN | 15.8 ± 1.0 | 16.0 ± 0.7 | 21.7 ± 1.4 |

[a]Food consumption was measured daily throughout the experiment. Results depicted represent the mean ± s.e.m. for food consumption of each group on the week listed.

**Table 3** Serum cholesterol levels in rats fed various polyunsaturated fat diets with and without neomycin

| Diet | Serum cholesterol (mg 100 $ml^{-1}$) |
|---|---|
| 20% Safflower oil | 87 ± 11[a] |
| 20% Safflower oil + neomycin | 51 ± 8[b] |
| 20% Safflower oil + cholesterol + cholic acid | 340 ± 63 |
| 20% Safflower oil + cholesterol + cholic acid + neomycin | 186 ± 20[b] |

[a]Mean ± s.e.m taking five rats per dietary group. [b]$P < 0.025$ (*t*-test) compared to corresponding dietary group without neomycin.

### Serum cholesterol levels

These were determined by the method of Carpenter et al (1957).

### Method for quantitation of faecal neutral and acid sterols

Twenty-four-hour stool output was collected from rats of each dietary group every 90 days. The faeces were homogenized with a measured volume of methanol, and subsequently lyophilized. For quantitation of neutral and acidic sterols, we used the techniques of Miettinen et al (1965) for neutral sterol analysis, and those of Grundy et al (1965), Deschner et al (1977) and Raicht et al (1975) for acid sterol analysis, with modifications made in our laboratory. The modifications included the addition of [$^3$H(G)] glycocholic acid (New England Nuclear, Boston, MA, USA) instead of [24-$^{14}$C] deoxycholic acid as a recovery standard to each sample at the beginning of the analysis to correct for incomplete recoveries during extraction and thin layer chromatography. Since bile acids are excreted as conjugates in the faeces, the addition of radioactive bile acid conjugate for the evaluation of recovery was most appropriate for precise measurements of these bile acids. The modifications also included the use of 5β-cholanic acid methyl ester and coprostanol as internal standards for quantitations of neutral and acid sterols, respectively, instead of 5α-cholestane. Since 5α-cholestane comes almost with the solvent peak, its use as an internal standard in the gas chromatographic analysis of faecal neutral and acid sterols may lead to erroneous results. 5β-cholanic acid methyl ester and coprostanol are suitable in this respect, since they meet all the criteria of internal standard. The methylation solution for faecal acid sterols before thin layer chromatography was prepared by the slow addition of freshly distilled acetyl chloride to 100 ml of lipopure methanol. These were purchased as instant methanolic HCl kit from Applied Science Laboratories, Inc (Pennsylvania, USA).

*© 1999 Cancer Research Campaign* 

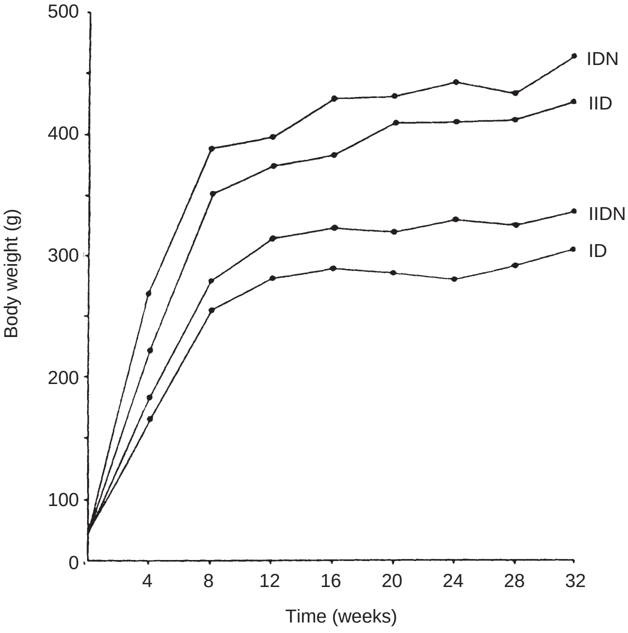

**Figure 1** Growth of DMH-administered rats fed various polyunsaturated fat diets with or without neomycin. ID: 20% safflower oil; IDN: 20% safflower oil + neomycin; IID: 20% safflower oil + cholesterol + cholic acid; IIDN: 20% safflower oil + cholesterol + cholic acid + neomycin. Values are mean ± s.e.m. starting with 20 rats per dietary group. IDN > IIDN, IID > ID and IID > IIDN, $P < 0.05$. IDN > ID, $P < 0.01$ (*t*-test)

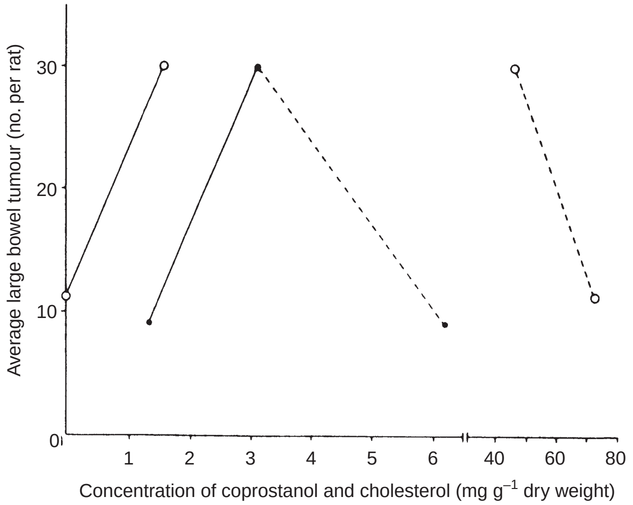

**Figure 2** Correlation of faecal coprostanol and cholesterol with large bowel tumorigenesis. ●–●: correlation of coprostanol between ID and IDN; ○–○: correlation of coprostanol between IID and IIDN; ●.....●: correlation of cholesterol between ID and IDN; ○.....○: correlation of cholesterol between IID and IIDN

Both the neutral and acid sterols were quantitated with a Hewlett Packard Model 873 gas chromatograph instrument on a 6-foot column packed with 3% ov-101 on 100–120 mesh Supelcoport. Column temperature was 260°C and that of the inlet and detector temperature was 280°C. All the analyses were performed in duplicate leading to more reliable results.

### RESULTS

Figure 1 illustrates the weight gained by the rats in different groups during the course of the study. It is evident that rats on IDN diet gained significantly more weight than those fed IIDN diet ($P < 0.05$). Also, an increase in growth rate has been exhibited by rats on IID diet from those on ID diet ($P < 0.05$). Within dietary groups, addition of neomycin to basal diet (ID) has shown an increase in weight ($P < 0.01$), while addition of neomycin to high cholesterol diet (IID) exhibited a decrease in weight ($P < 0.05$).

Food consumption of animals fed the various diets presented in Table 2 shows that up to the first 4 weeks, rats on IDN diet consumed significantly more than those on IIDN diet ($P < 0.01$). Same trend has been observed between rats fed high cholesterol diet and basal diet (IID > ID, $P < 0.01$). But from 4 to 16 weeks, all the rats consumed the same amount of food. However, from 16 to 32 weeks, rats fed neomycin with or without cholesterol consumed more than those fed diets without neomycin (IDN > ID, IIDN > IID, $P < 0.05$).

Table 3 illustrates the serum cholesterol levels of rats of different dietary regimens. It appears that rats fed high cholesterol diets have significantly increased serum cholesterol levels than those fed diets without cholesterol ($P < 0.01$). However, within each dietary group, addition of neomycin has exhibited a significant lowering of serum cholesterol levels ($P < 0.025$).

**Table 4** Large bowel and small bowel tumour incidence and average tumour number in DMH-treated rats fed various polyunsaturated fat diets with and without neomycin

| Diet | No. of rats | Incidence of small bowel tumours | Average no. of small bowel tumours per rat | Incidence of large bowel tumours | Average no. of large bowel tumours per rat |
|---|---|---|---|---|---|
| ID | 20 | 25% | 0.35 ± 0.40 | 60% | 0.90 ± 0.30 |
| IDN | 8 | 25% | 0.50 ± 0.20 | 100%[a] | 3.00 ± 0.20 |
| IID | 20 | 20% | 0.35 ± 0.10 | 100% | 3.00 ± 0.70[b] |
| IIDN | 8 | 37.5% | 0.50 ± 0.20 | 75%[a] | 1.13 ± 0.30[c] |

[a]As compared with corresponding dietary group without neomycin, $P < 0.01$ ($\chi^2$ test). [b]As compared to dietary group without neomycin and cholesterol, $P < 0.01$ (ANOVA). [c]As compared to dietary group with neomycin and without cholesterol, $P < 0.01$ (ANOVA).

Table 4 represents the incidence and numbers of large and small bowel tumours in DMH-treated rats fed different diets with or without neomycin. Rats fed the basal diet only (ID) have been found to have the lowest incidence and smallest number of large bowel tumours, which has increased on addition of neomycin. Supplementing the basal diet with cholesterol and cholic acid (IID) also augmented large bowel tumorigenesis. However, addition of neomycin to this group reduced the incidence and number of large bowel tumours. No significant differences were noted in either the incidence or the number of small bowel tumours among the groups.

Faecal excretion of neutral and acid sterols in DMH-treated rats fed various safflower oil diets with and without neomycin (illustrated in Table 5) shows significant increase in cholesterol excretion in cholesterol feeding rats than in non-cholesterol feeding ones (IID > ID, $P < 0.01$). This trend has also been shown within cholesterol feeding groups (IIDN > IID, $P < 0.01$). The concentration of faecal coprostanol was significantly higher in rats fed 20% safflower oil and neomycin (IDN) than any other dietary groups ($P < 0.01$), and the concentration was found to be least in rats on

© 1999 Cancer Research Campaign

**Table 5** Faecal excretion of neutral and acid sterols in DMH-treated rats fed various dietary regimens with and without neomycin

| | | Faecal neutral sterols (mg $g^{-1}$ dry weight) | | | Faecal acid sterols (mg $g^{-1}$ dry weight) | | | |
|---|---|---|---|---|---|---|---|---|
| **Diet** | **No. of rats** | **Cholesterol** | **Coprostanol** | **Total** | **Cholic** | **Deoxycholic** | **Lithocholic** | **Total** |
| ID | 4 | 6.19 ± 2.89[d] | 1.36 ± 0.30 | 7.55 ± 3.07 | 1.08 ± 0.76 | 1.99 ± 1.45 | 0.56 ± 0.30 | 3.63 ± 2.43 |
| IDN | 7 | 3.14 ± 0.46 | 3.13 ± 0.73[c] | 6.27 ± 0.41 | 0.21 ± 0.07 | 0.73 ± 0.14 | 0.53 ± 0.06 | 1.47 ± 0.18 |
| IID | 7 | 46.82 ± 2.07[a] | 1.57 ± 0.89 | 49.40 ± 2.81[a] | 5.85 ± 3.09 | 12.43 ± 3.02 | 3.00 ± 0.92[a] | 21.28 ± 0.83[a] |
| IIDN | 4 | 72.51 ± 1.70[a,b] | 0.00[c,e] | 72.1 ± 1.70[a,b] | 31.94 ± 5.72[b] | 0.00[e] | 2.14 ± 0.62[a] | 34.08 ± 5.14[a,b] |

[a]As compared to dietary groups without cholesterol, $P < 0.01$ (ANOVA). [b]As compared to dietary group without cholesterol, $P < 0.01$ (*t*-test). [c]As compared to dietary groups without neomycin, $P < 0.025$ (*t*-test). [d]Mean ± s.e.m. [e]Peak non-detectable in the gas chromatogram.

IIDN diet. Faecal excretion of cholic acid has been found to be higher in rats fed high cholesterol diet with neomycin (IIDN) than in those on other dietary regimens ($P < 0.01$). The concentration of deoxycholic acid in the faeces was higher in rats on IID diet than any other dietary groups ($P < 0.01$), while faecal excretion of lithocholic acid remained almost the same comparing ID with IDN and IID with IIDN.

From Tables 4 and 5, it is apparent that with increase in number of tumours on addition of neomycin to the rats fed the basal diet only (ID), concentration of coprostanol increased significantly while that of cholesterol decreased (ID vs IDN, $P < 0.01$). However, this trend has reversed in the cholesterol-fed groups of rats, where addition of neomycin to group IID dietary regimen decreased large bowel tumorigenesis, but concentration of faecal coprostanol also decreased with increase in faecal cholesterol concentration (IID vs IIDN, $P < 0.01$). Thus the positive and negative correlations of coprostanol and cholesterol, respectively, with the incidence of large bowel tumorigenesis in rats fed the four dietary regimens are illustrated in Figure 2.

### DISCUSSION

The long-term objective of this laboratory is to identify nutritional factors that contribute to the high incidence of colon cancer in Western culture. In the current study, 20% safflower oil diet was used with or without cholesterol, in order to have a correlation between dietary cholesterol and large bowel tumorigenesis. Our early studies utilizing atherogenic diets and DMH for tumour induction have implied that cholesterol is the key issue, whether it is retained in systemic and enterohepatic circulation, or removed and subjected to microbial modification in colon (Broitman et al, 1977; Kraus et al, 1987; Fabricant and Broitman, 1990). Thus, increasing the quantity of cholesterol to the large bowel by increased intake of dietary cholesterol might contribute to the enhanced development of large bowel tumour. The augmentation of large bowel tumorigenesis on addition of cholesterol to 20% polyunsaturated fat diet (ID vs IID, $P < 0.01$, $\chi^2$ test) in this study has confirmed the above findings (Table 4).

Lowering of serum cholesterol and shunting it out through the gut via a hypocholesterolaemic drug has been reported to enhance colon carcinogenesis. Studies with clofibrate, cholestyramine (Steiner et al, 1991), neomycin (Broitman et al, 1960; Sedaghat *et al*, 1975; Kesaniemi and Miettinen, 1991) etc. have been suggestive enough to promote concern regarding the effects of lowering serum cholesterol with drugs to the development of gastrointestinal tract neoplasms. Many hypolipidaemic agents are associated with increased faecal bile acids and neutral sterol excretion, and have serum cholesterol lowering effects. Neomycin is one such agent which lowers serum cholesterol level and inhibits cholesterol absorption in colon of man (Kesaniemi and Miettinen, 1991). The data presented in Table 3 show that addition of neomycin to 20% safflower oil diets with or without cholesterol has lowered the serum cholesterol levels significantly (ID vs IDN, IID vs IIDN, $P < 0.025$, *t*-test). In our study, addition of neomycin to 20% safflower oil diet (ID) enhanced the incidence of large bowel tumours (Table 4). This result is in agreement with earlier reports suggesting a positive correlation of serum cholesterol-lowering effect of neomycin with development of colon tumours. However, there was no change in small bowel tumour numbers with addition of neomycin to the rats fed basal diet (ID vs IDN). Although there has been an increase in small bowel tumorigenesis on addition of neomycin to cholesterol-feeding groups (IID vs IIDN), the increase was not significant.

It has been difficult to ascertain whether bile acids or neutral sterols are responsible for augmentation of tumorigenesis. In our laboratory it is believed that the neutral sterols excreted in the large-bowel lumen might in some manner augment this process. The use of neomycin affords a particular advantage in that its hypocholesterolaemic effect is almost exclusively related to an increased output of faecal neutral sterols. Thus, it should be possible to ascertain if increased tumorigenesis occurs in animals fed neomycin compared to animals not fed neomycin, since neomycin alters the neutral and acid sterol concentrations in gut and relates them to alterations of tumour numbers. Such an event would be associated with increased levels of neutral sterols and this would implicate neutral sterols as a participant in large bowel tumorigenesis rather than bile acids. In our experiment, neomycin enhanced incidence of large bowel tumours significantly (Table 4) when added to the dietary group ID, which has been accompanied by a significant increase in faecal coprostanol (Table 5). Thus, our data could satisfactorily correlate the enhanced excretion of faecal neutral sterol coprostanol to the augmented large-bowel tumorigenesis (ID vs IDN, correlation coefficient, $r = +1$). In the second dietary group, IID, effect of neomycin apparently did not seem to show the same pattern with respect to its control. The incidence of large bowel tumorigenesis was less by 25% (IID vs IIDN, $P < 0.01$, $\chi^2$ test) when neomycin was added to the cholesterol-supplemented diet (Table 4). Therefore, neomycin augmented tumorigenesis when the polyunsaturated fat diet contained no cholesterol, and decreased incidence of large bowel tumours when the same diet was supplemented with cholesterol. This indicated a complex interaction of neomycin with the cholesterol metabolism in gut. Presence of neomycin in conjunction with dietary cholesterol might have some regulatory role to play in inhibiting large

*© 1999 Cancer Research Campaign*

bowel tumorigenesis in rats. This might be possible either by altering the sterol metabolism in gut, or by making cholesterol unavailable to the large bowel lumen by inhibiting its absorption in colon or by feedback inhibition of cholesterol synthesis in gut. The exact mechanism is yet to be established. The large bowel flora might have an important role to play in this regard.

From the faecal excretion data (Table 5), it has been found that faecal cholesterol levels were always less in each dietary group where there were more tumours. Thus, the amount of faecal cholesterol has a negative correlation with tumour number (correlation coefficient, $r = -1$). However, faecal coprostanol excretion was consistently high when there were more tumours, which was satisfactorily shown in Figure 2. These observations could give credence to the concept developed by earlier workers (Reddy et al, 1977; Carroll, 1987; Reddy, 1992) that due to the dietary lipid-induced alteration of the faecal flora, the sterols modified in gut might act as carcinogens or co-carcinogens. Thus, enhanced faecal excretion of coprostanol and diminished excretion of faecal cholesterol with augmented large bowel tumorigenesis in this study might imply increased activity of the intestinal flora in transformation of cholesterol and/or its metabolites in gut. Like cholesterol, cholic acid also showed negative correlation with large bowel tumorigenesis. The reason could not be ascertained satisfactorily. Cholic acid might have contributed to the bile acid pool in the animals. There is no evidence that cholic acid may act as a promoter or co-carcinogen. The concentrations of the secondary bile acids, deoxycholic and lithocholic acids did not seem to contribute to large bowel tumorigenesis, as shown in the data in Table 5.

In view of the above findings, it can be concluded that, in DMH-fed rats fed polyunsaturated fat diets supplemented with neomycin and cholesterol, coprostanol might have some contributions in augmenting large bowel tumorigenesis. All other neutral and acidic sterols seem to have no participation. The probable role of coprostanol might be taken as an important factor, and regulation of cholesterol metabolism in gut and variations in bacterial population responsible for the alteration of the faecal sterols might provide some preliminary insight in prevention of colon cancer.

### ACKNOWLEDGEMENT

This project was partly funded by NIH, USA.

### REFERENCES

Broitman SA, Kinnear DG, Gottlieb LS, Bezman AL, Vitale JJ and Zamcheck N (1960) Effect of neomycin alteration on the rat intestinal flora on serum cholesterol and valvular sudanophilia. *J Lab Clin Med* **55**: 55–59

Broitman SA, Vitale JJ, Vavrousek-Jakuba E and Gottlieb LS (1977) Polyunsaturated fat, cholesterol and large bowel tumorigenesis. *Cancer* **40**: 2455–2463

Cannizzo F Jr and Broitman SA (1989) Postpromotional effects of dietary marine or safflower oils on large bowel or pulmonary implants of CT-26 in mice. *Cancer Res* **49**: 4289–4294

Carpenter K, Gotsis A and Hegsted DM (1957) Estimation of total cholesterol in serum. *Clin Chem* **3**: 233–288

Carroll KK (1987) Summation: which fat/how much fat – animals. *Prev Med* **16**: 510–515

Cerda SR, Wilkinson J IV and Broitman SA (1995) Regulation of cholesterol synthesis in four colonic adenocarcinoma cell lines. *Lipids* **30**: 1083–1092

Cohen BI, Raicht RF, Deschner EE, Fazzini E, Takahashi M and Sarwal A (1978) Effects of bile acids on induced colon cancer in rats. *Proc Am Assoc Cancer Res* **19**: 48

Corpet DE, Bellier R, Petrowitsch S and Vigouroux Y (1994) Digestion and fermentation of proteins in rats fed keratin, albumin, cooked casein and antibiotics. *Reprod Nutr Dev* **34**: 57–64

Deschner EE, Winawer SJ, Long FC and Boyle CC (1977) Early detection of colonic neoplasia in patients at high risk. *Cancer* **40**: 2625–2631

Doll R (1969) The geographical distribution of cancer. *Br J Cancer* **23**: 1–8

Fabricant M and Broitman SA (1990) Evidence for deficiency of low density lipoprotein receptor on human colonic carcinoma cell lines. *Cancer Res* **50**: 632–636

Grundy SM, Ahrens EH and Miettinen TA (1965) Quantitative isolation and gas-liquid chromatographic analysis of the total bile acids. *J Lipid Res* **6**: 397–410

Haenszel W, Berg JW, Kurihara M and Locke FB (1973) Large bowel cancer in Hawaiian Japanese. *J Natl Cancer Inst* **51**: 1965–1799

Kesaniemi YA and Miettinen TA (1991) Inhibition of cholesterol absorption by neomycin, benzodiazepine derivatives and ketoconazole. *Eur J Clin Pharmacol* **40**: S65–S67

Kraus LJ, Williams RM and Broitman SA (1987) T-cell mitogenesis and natural killer cell activity in colonic tumor-bearing and nontumor-bearing rats fed diets high in lipid with and without cholesterol. *Nutr Cancer* **9**: 159–170

Lindner MA (1991) A fish oil diet inhibits colon cancer in mice. *Nutr Cancer* **15**: 1–11

Miettinen TA, Ahrens EH and Grundy SM (1965) Quantitative isolation and gas-liquid chromatographic analysis of total dietary and faecal neutral steroids. *J Lipid Res* **6**: 411–424

Narisawa T, Takahashi M, Kotanagi H, Kusaka H, Yamazaki Y, Koyama H, Fukaura Y, Nishizawa Y, Kotsugai M and Isoda Y (1991) Inhibitory effect of dietary perilla oil rich in the n-3 polyunsaturated fatty acid alpha-linolenic acid on colon carcinogenesis in rats. *Jpn J Cancer Res* **82**: 1089–1096

Narisawa T, Fukaura Y, Yazawa K, Ishikawa C, Isoda Y and Nishizawa Y (1994) Colon cancer prevention with a small amount of dietary perilla oil high in alpha-linolenic acid in an animal model. *Cancer* **73**: 2069–2075

Ochiai M, Nakagama H, Watanabe M, Ishiguro Y, Sugimura T and Nagao M (1996) Efficient method for rapid induction of aberrant crypt foci in rats with 2-amino-1-methyl-6-phenylimidazo [4,5-b] pyridine. *Jpn J Cancer Res* **87**: 1029–1033

Raicht RF, Cohen BI, Shefer S and Mosbach EH (1975) Sterol metabolism in the rat. Effect of dietary cholesterol and β-sitosterol and sterol balance and rate limiting enzymes of sterol metabolism. *Biochim Biophys Acta* **388**: 374–384

Reddy BS (1992) Dietary fat and colon cancer: animal model studies. *Lipids* **27**: 807–813

Reddy BS, Martin CW and Wynder EL (1977) Faecal bile acids and cholesterol metabolites of patients with ulcerative colitis, a high risk group for development of colon cancer. *Cancer Res* **37**: 1697–1701

Sedaghat A, Samuel P, Crouse JR and Ahrens EH (1975) Effects of neomycin on absorption, synthesis and/or flux of cholesterol in man. *J Clin Invest* **55**: 12–21

Steiner A, Weisser B and Vetter W (1991) A comparative review of the adverse effects of treatments for hyperlipidaemia. *Drug Saf* **6**: 118–130

Wynder EL (1975) The epidemiology of large bowel cancer. *Cancer Res* **35**: 338–3394

 © 1999 Cancer Research Campaign
